# Infective Endocarditis, a Current Perspective: Clinical and Epidemiological Profile in a High-Volume Chilean Tertiary Centre Between 2021–2023

**DOI:** 10.3390/pathogens15070727

**Published:** 2026-07-10

**Authors:** Ignacio Hernan Pineda Etcheber, Cheryld Mutel Gonzalez, Javiera Antonia Bascuñan Maiz, Antonia Cesped Astete, Mauricio Soto Vasquez

**Affiliations:** 1 Internal Medicine Department, Universidad de La Frontera, Temuco 4781218, Chile; ignacio.pineda@asur.cl (I.H.P.E.); cheryd.mutel@ufrontera.cl (C.M.G.); 2 Intensive Care Unit, Complejo Asistencial de Padre Las Casas, Padre Las Casas 4850000, Chile; 3 Cardiovascular Centre, “Dr. Hernán Henríquez Aravena” Hospital, Temuco 4781151, Chile; 4 Medicine School, Universidad de La Frontera, Temuco 4781218, Chile

**Keywords:** infective endocarditis, epidemiology, *Streptococcus gallolyticus*, mortality, Chile

## Abstract

Infective endocarditis (IE) is a severe pathology with recent changes in its epidemiological profile, characterised by older patients with more comorbidities. The objective of this study is to describe the clinical and microbiological characteristics, as well as potential factors associated with mortality, of patients with IE in a tertiary academic centre. **Material and Methods:** Descriptive, retrospective, and observational study of patients over 18 years of age with a confirmed diagnosis of IE, conducted between 2021 and 2023 at the Dr Hernán Henríquez Aravena Hospital in Temuco, Chile. Biodemographic variables, risk factors, microbiology, echocardiographic findings, and complications were analysed using descriptive statistics and logistic regression models. **Results:** 119 patients were included (average age 60 years; 65.5% male; 28.5% rural). The most frequent risk factors were arterial hypertension (55%) and diabetes mellitus (29%). 18% were on haemodialysis (HD). Microbiological isolation was achieved in 78.1% of cases, with *Streptococcus gallolyticus* the most frequent isolate (16.8%), followed by *Staphylococcus aureus* (15.1%) and coagulase-negative *Staphylococcus* (15.1%). Complications were present in 69% of cases, mainly emboli (43%) and septic shock (23%)—59.6% required surgery. Global mortality was 44.5%, with a decreasing annual trend (from 58% in 2021 to 33% in 2023). Comorbidities and complications independently associated with mortality were chronic renal failure on HD (OR 5.76; *p* = 0.001), heart failure (OR 3.13; *p* = 0.025), and septic shock (OR 3.31; *p* = 0.016). **Conclusions:** IE in this centre presents an aggressive profile and a high burden of comorbidities. The prevalence of *S. gallolyticus* is a notable regional observation not reported in similar populations. Mortality remains high, with an improving trend.

## 1. Introduction

Infective endocarditis (IE) is an infectious disease characterised by endocardial involvement by pathogenic agents, which usually manifests as valvular involvement; historically, it has been associated with severe clinical compromise and high mortality [1].

The incidence of this pathology has increased, with a global estimate of 13.8 cases per 100,000 patients per year [1]. At the national level in Chile, the latest data indicate an incidence of 2–3 cases per 100,000 inhabitants [2]. However, the profile of patients suffering from IE has modified over time as rheumatic disease has regressed; presentation ages are increasingly higher, and there is a high association with traditional cardiovascular risk factors and intravenous drug use [1,3].

From a microbiological perspective, the main causative agent worldwide remains Staphylococcus aureus [3], which has also been reported in multiple Chilean series [4,5,6]. However, significant differences exist in the population of southern Chile, as observed in a series described over a decade ago by Stockins et al. [7]; in their series of 107 patients identified in our tertiary hospital centre, the most frequently identified agent was *Streptococcus viridans*, isolated in 31% of the evaluated patients. However, due to changing population characteristics, emerging causative agents and novel resistance patterns are increasingly being described, warranting the constant updating of microbiological data to adapt to these conditions [8].

Regarding mortality, it remains high along with the change in clinical profile (better diagnostic and therapeutic resources, but older patients with greater comorbidities and more complex valvular involvement); reports in Chilean hospitals from the 2010s onwards place it between 20% and 45%, depending on the centre [9], while internationally it is estimated between 19% and 25% [3].

The objective of the present study is to describe the up-to-date clinical and epidemiological characteristics of adult patients with IE at the Hospital Hernán Henríquez Aravena (HHHA) in Temuco, a high-complexity tertiary academic centre in Chile, and to evaluate potential associations between determinants that may influence the high mortality of this condition.

## 2. Materials and Methods

This is a descriptive, retrospective, and observational study. Patients aged 18 or older with a confirmed discharge diagnosis of IE according to modified Duke criteria [10] who were admitted to the HHHA between January 2021 and December 2023 were included. Exclusion criteria included cases in which the IE diagnosis could not be confirmed due to incomplete or insufficient clinical records, as well as patients with non-infective endocarditis (non-bacterial thrombotic or marantic endocarditis). Cases with missing primary data or unresolved discrepancies were excluded to ensure data integrity. Collected variables included biodemographic data, risk factors, clinical characteristics at admission, local complications (presence of fistula, abscess, perforation or pseudoaneurysm), imaging and microbiologic findings and clinical complications such as the presence of embolism of new onset (with radiological confirmation by computed tomography or magnetic resonance imaging); septic shock (according to Sepsis-3 guidelines), acute heart failure (New York Heart Association [NYHA] functional classification IV of new onset or acute clinical worsening requiring intravenous diuretic therapy), and cardiogenic shock (acute heart failure with signs of systemic hypoperfusion).

The study protocol was approved by the scientific ethics committee of the Araucanía Sur Health Service and has the corresponding authorisation from our centre. Data were collected in an anonymised database and analysed by a blinded external statistician. Statistical analysis was performed using Stata 17.0. Categorical variables were compared using the chi-square test or Fisher’s exact test, while continuous variables were analysed using Student’s *t*-test, as appropriate. Variables associated with mortality in the univariate analyses and considered clinically relevant were included in a multivariate logistic regression model to identify independent predictors of in-hospital mortality. To reduce the risk of overfitting, the number of variables included in the final multivariate model was restricted according to the number of observed mortality events.

## 3. Results

A total of 125 patients were initially screened; 6 patients were excluded due to incomplete clinical registries or unresolved discrepancies in primary data, yielding a final analytical cohort of 119 patients who met the inclusion criteria. The baseline characteristics of the population are detailed in Table 1. Baseline clinical and biodemographic characteristics are detailed in Table 1; an advanced age profile, male predominance, and a substantial burden of chronic cardiovascular and renal comorbidities characterised the cohort.

### 3.1. Clinical Presentation

Clinical manifestations at the time of diagnosis are compiled in Table 2. The presentation was heavily dominated by objective fever and detectable cardiac murmurs, frequently accompanied by non-specific constitutional symptoms, dyspnea, and acute neurological deficits.

### 3.2. Echocardiographical Features

Echocardiographic findings are summarised in Table 3. Left ventricular systolic function was generally preserved across the cohort. Valvular vegetations predominantly affected left-sided structures, with a high prevalence of severe regurgitant lesions and multi-valvular involvement.

### 3.3. Microbiological Diagnosis

The microbiological profile is provided in Table 4. Pathogen isolation was successful in the majority of cases, driven primarily by automated blood cultures, with additional identification via valve tissue culture, universal PCR, and serology in culture-negative cases. Systematic antimicrobial susceptibility testing (AST) was consistently available and routinely performed for all positive cultures to determine specific resistance profiles, in compliance with current Clinical and Laboratory Standards Institute (CLSI) guidelines. *Streptococcus gallolyticus* was the most common isolate, followed closely by *Staphylococcus aureus* and coagulase-negative *Staphylococci*. Notably, there were no cases of methicillin-resistant *Staphylococcus aureus* or vancomycin-resistant *Enterococci species*.

### 3.4. Evolution and Complications

In-hospital clinical complications occurred in 69.7% of the cohort, as documented in Table 5. Embolic events (predominantly cerebral) and septic shock represented the most frequent complications. Valvular complications occurred in 27.0% of cases, and severe clinical deterioration necessitated surgical management in 59.7% of patients. Exploratory univariate analysis suggested associations between diabetes and coronary artery disease with cardiogenic shock, and prior pulmonary disease with septic shock; however, these subgroup associations must be interpreted with caution due to low subgroup event counts and wide confidence intervals, which indicate limited statistical stability.

### 3.5. Mortality

Annual mortality showed a progressive decline, decreasing from 58.0% in 2021 to 44.0% in 2022 and 33.0% in 2023. Univariate predictors of higher in-hospital mortality included arterial hypertension and end-stage renal disease on haemodialysis (Table 6). Advanced age showed a non-significant upward trend (OR 1.02 per additional year; *p* = 0.061).

Univariate analysis of clinical complications identified septic shock and cardiogenic shock as significant predictors of mortality. In the final multivariate logistic regression model (Table 7), acute heart failure and septic shock remained independently associated with in-hospital mortality. In contrast, cardiogenic shock exhibited model instability due to the small sample size (*n* = 8). Embolism was not significantly linked to survival outcomes (*p* = 0.184).

The distribution of complications by pathogen is presented in Table 8. Isolation of Staphylococcus aureus, coagulase-negative Staphylococci, and non-viridans Streptococci was significantly associated with the development of septic shock. Conversely, infection with the Streptococcus viridans subgroup was associated with a significantly lower risk of in-hospital mortality. No stable associations were found between specific pathogens and embolic phenomena, acute heart failure, or cardiogenic shock.

## 4. Discussion

The series presented in this article, with 119 patients, is among the largest reported in Chile, with an average of 40 cases per year, significantly exceeding the incidence reported at other Chilean centres of similar size. The prior largest case series was a 506-case report spanning 20 years across 37 metropolitan-area hospitals [2]. This higher case burden can be explained by our academic centre’s large area of influence, comprising 89.544 km^2^, and by its role as the sole public cardiac surgery centre for 2 million inhabitants. This extensive tertiary referral profile creates a substantial selection and referral bias, concentrating highly complex, severe, and late-stage cases referred from secondary and primary networks across the region, thereby inherently limiting the direct generalizability of our findings to less complex or non-referral hospital populations.

Our cohort reflects the global shift in IE towards an older population with a higher burden of comorbidities and traditional risk factors, aligning with European series, which report an average age of 60 years and a 2:1 male-to-female ratio [11], in contrast with North American series, where the average age is much lower [12], partly influenced by intravenous drug use, which was completely absent in our setting. This higher average age reflects the global change IE has undergone, shifting towards an increasingly older population with greater presence of comorbidities and traditional cardiovascular risk factors [3,11,12].

Regarding aetiology, *Streptococcus gallolyticus* emerged as the most frequent isolate (16.8%). This high incidence of valvular compromise has not been previously described in the Chilean population, even in the most recent series [13]. It stands out as a unique finding in our regional series. While our regional reference population exhibits high baseline rates of rurality (29.1%) and poverty (19.8%) according to the 2022 CASEN survey [14], any association between these socioeconomic factors and the distribution of specific pathogens remains strictly speculative since our retrospective study design did not collect individual-level data regarding household sanitary conditions, agricultural exposures, or specific social determinants. Notably, in our series, there is an absence of MRSA or VRE as aetiological agents, which could be partly explained by the lack of intravenous drug use in our population.

The overall in-hospital mortality rate of 44.5% observed in this study is markedly higher than that reported in contemporary international reports, which generally range between 19% and 25% [3]. This stark discrepancy cannot be attributed solely to patient comorbidities and requires a deeper analysis of structural and systemic factors. First, the massive geographical catchment area of 89,544 km^2^ poses severe logistical barriers, resulting in significant diagnostic and surgical delays during long-distance transfers. This delay is objectively demonstrated by the exceptionally high rate of embolic complications at diagnosis (42.8%), far exceeding the Latin American (27%) and European (20%) benchmarks [15,16,17], indicating advanced, aggressive valvular destruction before tertiary intervention can occur. Second, the study window (2021–2023) directly overlapped with the severe disruptions of the COVID-19 pandemic. During 2021 and 2022, critical shortages of intensive care beds, delays in semi-urgent cardiac surgeries, and restricted outpatient diagnostic capabilities substantially prolonged the time to definitive surgical intervention, driving the excess mortality seen in the earlier years of the registry (58% in 2021). Lastly, because our centre concentrates all public cardiac surgery cases for 2 million people, there is a clear severity bias, where only the most critically ill or hemodynamically compromised patients are selected for transfer, compounding the observed mortality.

Weaknesses: This study has several important limitations that warrant attention. First, its single-centre, retrospective design introduces inherent referral and selection biases, limiting the generalizability of the results to the broader population or non-tertiary centres. Second, some subgroup analyses showed wide confidence intervals due to the limited number of events in specific complications, particularly cardiogenic shock; therefore, these findings should be interpreted with caution and validated in larger, multicenter cohorts. Third, as previously noted, the lack of granular data on individual socioeconomic factors, systematic gastrointestinal screenings, and antimicrobial resistance profiles prevents a comprehensive characterisation of the clinical–microbiological landscape. These limitations underscore the urgent need for a robust, prospective, multicentre national registry to characterise infective endocarditis in Chile fully.

## 5. Conclusions

Through this series, we expose the reality of this disease in our tertiary centre, where IE presents with a more aggressive profile and a higher rate of complications requiring surgical intervention, posing the challenge of intensifying efforts to achieve the earliest possible diagnosis. While our findings show significant associations between high baseline comorbidity burden and poor clinical outcomes, the retrospective and observational nature of this study precludes the establishment of definitive causal mechanisms or broad population extrapolations. Future strategies should focus on establishing prospective national registries to confirm these associative trends, improve diagnostic timeliness, and systematically investigate the unexpectedly high prevalence of *Streptococcus gallolyticus*, including its potential association with gastrointestinal portals of entry.

## Figures and Tables

**Table 1:** Baseline Characteristics.

Variable	N (%)
Age (average/median)	59.7/60
Male sex	78 (65.5)
Rurality	34 (28.6)
Smoking	14 (11.8)
Alcoholism	33 (27.7)
Intravenous drug use	0 (0)
Previous dental procedure	8 (6.7)
Hypertension	66 (55.5)
Type 2 Diabetes mellitus	35 (29.4)
End-stage renal disease	22 (18.5)
Cirrhosis	9 (7.6)
Pulmonary disease	6 (5.0)
Heart failure	24 (20.2)
Coronary artery disease	7 (5.9)
Congenital heart disease	3 (2.5)
Presence of an intracardiac device	6 (5.0)
Presence of prosthetic valve	11 (9.2)

**Table 2:** Clinical manifestations at the time of diagnosis.

Variable	N (%)
Presence of a cardiac murmur	95 (79.8)
Fever	82 (68.9)
Malaise	61 (51.3)
Dyspnea	48 (40.3)
Acute neurological deficit (any)	24 (20.2)
Gastrointestinal symptoms	22 (18.5)
Musculoskeletal pain	18 (15.1)
Back pain	17 (14.3)
Weight loss	14 (11.8)

**Table 3:** Echocardiographical findings.

Variable	
Left ventricular ejection fraction LVEF, average (median)	57% (58%)
Location of vegetations	N (%)
Aortic valve	69 (58.0%)
Mitral valve	50 (42.0%)
Intracardiac device lead	6 (5.0%)
Tricuspid valve	4 (3.4%)
Type and severity of valvular lesions	
Aortic regurgitation, any grade	69 (58.0%)
Severe aortic regurgitation	19 (16.0%)
Mitral regurgitation, any grade	62 (52.1%)
Severe mitral regurgitation	53 (44.5%)
Aortic stenosis, any grade	20 (16.8%)
Severe aortic stenosis	8 (6.7%)
Tricuspid regurgitation, any grade	6 (5.0%)
Severe tricuspid regurgitation	3 (2.5%)
Pulmonary regurgitation, any grade	1 (0.8%)
Presence of more than 1 valvular defect	55 (46.2%)

**Table 4:** Microbiological findings.

Microorganism Identified	N (%)
*Streptococcus gallolyticus* (formerly *Streptococcus bovis*)	20 (16.8%)
*Staphylococcus aureus*	18 (15.1%)
Coagulase-negative *Staphylococci*	18 (15.1%)
*Streptococcus viridans*	12 (10.1%)
*Enterococcus* species	8 (6.7%)
Other *Streptococci*	5 (4.2%)
*Bartonella species*	2 (1.7%)
*Candida* species	2 (1.7%)
HACEK * group Gram-negative bacilli	1 (0.8%)
Other Gram-negative microorganisms	1 (0.8%)
Other **	6 (5.0%)
No microorganism identified	26 (21.8%)
Method of identification	
Blood cultures	84 (70.6%)
Valve tissue culture	3 (2.5%)
Polymerase chain reaction of valvular tissue	5 (4.2%)
Serology	1 (0.8%)

* HACEK group: *Haemophilus, Aggregatibacter, Cardiobacterium, Eikenella* and *Kingella* species. ** Other species: individual cases of micro-organisms not classically associated with endocarditis.

**Table 5:** Incidence of Clinical Complications.

Clinical Complications	
Any clinical complication	83 (69.7%)
Embolism at diagnosis	51 (42.9%)
Brain	31 (26.1%)
Spleen	18 (15.1%)
Kidney	7 (5.9%)
Lung	5 (4.2%)
Other	11 (9.2%)
Septic shock	27 (22.7%)
Acute heart failure	25 (21.0%)
Cardiogenic shock	8 (6.7%)
Requirement for surgical management	71 (59.7%)
Global In-hospital Mortality	53 (44.5%)

**Table 6:** Mortality according to baseline characteristics.

	Observed Mortality (%)	Odds-Ratio (95% CI)	*p*-Value
Sex			
Male	43.6	1.09 (0.51–2.33)	0.820
Female	46.3	Reference	-
Rurality	32.4	0.50 (0.21–1.10)	0.106
Smoking	42.9	0.67 (0.43–1.05)	0.082
Alcohol abuse	36.4	0.77 (0.57–1.05)	0.095
Prior dental procedure	25.0	0.39 (0.05–2.84)	0.364
Hypertension	54.5	2.54 (1.20–5.40)	0.014 *
Type 2 Diabetes Mellitus	54.3	1.75 (0.79–3.87)	0.169
End-stage renal disease	77.3	5.76 (1.96–16.95)	0.001 *
Liver cirrhosis	55.6	1.61 (0.41–6.33)	0.492
Lung disease	16.7	0.23 (0.02–2.07)	0.192
Chronic heart failure	58.3	2.01 (0.81–4.99)	0.132
Ischemic heart disease	71.4	3.33 (0.62–17.92)	0.161
Congenital heart disease	33.3	0.61 (0.05–6.98)	0.615
Prosthetic heart valve	63.6	3.03 (0.71–12.86)	0.133
Intracardiac pacing device	33.0	0.76 (0.13–4.34)	0.755

CI: Confidence Interval; * statistically significant result (*p* < 0.05).

**Table 7:** Multivariate analysis of mortality associations with clinical complications.

	Observed Mortality (%)	Odds-Ratio (95% CI)	*p*-Value
Acute heart failure	60.0	3.13 (1.16–8.45)	0.025 *
Septic shock	66.7	3.30 (1.24–8.75)	0.016 *
Cardiogenic shock	87.5	7.47 (0.80–66.96)	0.067
Embolism, any site	49.0	1.73 (0.77–3.91)	0.184

CI: Confidence Interval; * statistically significant result (*p* < 0.05).

**Table 8:** Complications by microorganism.

	Incidence (%)	Odds-Ratio (95% CI)	*p*-Value
Embolism			
Other *Streptococci*	80.0	6.40 (0.62–65.73)	0.095
*Staphylococcus aureus*	61.1	2.50 (0.73–8.63)	0.143
*Bartonella* species	50.0	1.60 (0.08–28.56)	0.750
*Candida* species	50.0	1.60 (0.08–28.56)	0.750
Other microorganisms	50.0	1.60 (0.26–9.53)	0.520
*Streptococcus viridans*	41.6	1.14 (0.28–4.60)	0.190
*Streptococcus gallolyticus*	40.0	1.06 (0.32–3.51)	0.110
Coagulase-negative *Staphylococci*	27.8	0.62 (0.16–2.25)	0.460
*Enterococcus* species	37.5	0.96 (0.18–4.92)	0.906
Septic shock			
Other *Streptococci*	60.0	18.0 (1.80–179.22)	0.014 *
Coagulase-negative *Staphylococci*	44,4	9.6 (1.72–53.40)	0.010 *
*Staphylococcus aureus*	33.3	6.0 (1.04–34.31)	0.044
*Streptococcus gallolyticus*	25.0	4.0 (0.68–23.29)	0.123
Other microorganisms	16.7	2.4 (0.18–31.88)	0.500
*Enterococcus* species	12.5	1.7 (0.13–21.81)	0.678
In-hospital mortality			
*Streptococcus viridans*	8.3	0.08 (0.008–0.694)	0.022 *
*Streptococcus gallolyticus*	35.0	0.46 (0.13–1.53)	0.206
Other *Streptococci*	40.0	0.57 (0.08–4.00)	0.573
*Staphylococcus aureus*	38.9	0.54 (0.16–1.85)	0.331
Coagulase-negative *Staphylococci*	55.6	1.07 (0.32–3.58)	0.911
*Enterococcus* species	75.0	2.57 (0.43–15.19)	0.297
*Candida* species	50.0	0.85 (0.04–15.22)	0.916
Other microorganisms	50.0	0.86 (0.14–5.06)	0.520

CI: Confidence Interval; * statistically significant result (*p* < 0.05).

## Data Availability

The data presented in this study are available upon request from the corresponding author, in accordance with Chilean Law protecting sensitive information.

## Abbreviations

The following abbreviations are used in this manuscript:

AST	Antibiotic susceptibility Testing
BC	Blood cultures
CLSI	Clinical and Laboratory Standards Institute
IE	Infective endocarditis
HHHA	“Dr. Hernán Henríquez Aravena” Hospital
LVEF	Left Ventricular Ejection Fraction
MO	Microorganism
MRSA	Methicillin-resistant *Staphylococcus aureus*
NYHA	New York Heart Association
OR	Odds-ratio
PCR	Polymerase chain reaction
VRE	Vancomycin-resistant *Enterococci*

## References

[1] Momtazmanesh S., Saeedi Moghaddam S., Malakan Rad E., Azadnajafabad S., Ebrahimi N., Mohammadi E., Rouhifard M., Rezaei N., Masinaei M., Rezaei N. (2022). Global, regional, and national burden and quality of care index of endocarditis: The global burden of disease study 1990–2019. Eur. J. Prev. Cardiol..

[2] Oyonarte M., Montagna R., Braun S., Rojo P., Jara J.L., Cereceda M., Morales M., Nazal C., Alonso F. (2012). Endocarditis infecciosa: Características clínicas, complicaciones y mortalidad en 506 pacientes y factores pronósticos de sobrevida a 10 años (1998–2008). Estudio cooperativo nacional en endocarditis infecciosa en Chile (ECNEI-2). [Infective endocarditis: Clinical characteristics, complications and mortality in 506 patients and 10-year survival pronostic factors (1998–2008). National cooperative study on infective endocarditis in Chile (ECNEI-2)]. Rev. Méd. Chile.

[3] Delgado V., Ajmone Marsan N., de Waha S., Bonaros N., Brida M., Burri H., Caselli S., Doenst T., Ederhy S., Erba P.A. (2023). 2023 ESC Guidelines for the management of endocarditis: Developed by the task force on the management of endocarditis of the European Society of Cardiology (ESC) Endorsed by the European Association for Cardio-Thoracic Surgery (EACTS) and the European Association of Nuclear Medicine (EANM). Eur. Heart J..

[4] Flores P., González N., Betancourt P., Berho J., Astudillo C., García C., Rojas J. (2017). Endocarditis Infecciosa: Caracterización clínica de la enfermedad. Revisión de casos de los últimos 5 años. [Infective endocarditis: Clinical characterization of the disease. Case review of the last 5 years]. Rev. Chil. Cardiol..

[5] Merello L., Rodrigo S.M., Elgueta G.F., González D., Elton V., Quiroz M., Pedemonte O., Aránguiz-Santander E. (2019). Sobrevida a 10 años de pacientes egresados luego de cirugía por endocarditis infecciosa en un hospital público. [10-year survival of patients discharged arter surgery by infective endocarditis in a public hospital]. Rev. Méd. Chile.

[6] Cruz J., Marín P., Migueles D. (2018). Endocarditis infecciosa en Hospital de Talca, período 1998–2015. [Infective endocarditis in the Talca Hospital, period 1998–2015]. Rev. Chil. Cardiol..

[7] Stockins B., Neira V., Paredes A., Castillo C., Troncoso A. (2012). Perfil clínico-epidemiológico de pacientes con endocarditis infecciosa, período 2003-2010 en el hospital de Temuco, Chile. [Clinical-epidemiological profile of patients with infective endocarditis, period 2003-2010 in the Temuco Hospital, Chile]. Rev. Méd. Chile.

[8] Reisinger M., Kachel M., George I. (2024). Emerging and Re-Emerging Pathogens in Valvular Infective Endocarditis: A Review. Pathogens.

[9] Del Castillo C., Tapia A., Begazo A., Oyonarte M. (2025). Clinical and epidemiological profile of infective endocarditis in Chile—A systematic review of descriptive analysis. Am. Heart J. Plus Cardiol. Res. Pract..

[10] Fowler V.G., Durack D.T., Selton-Suty C., Athan E., Bayer A.S., Chamis A.L., Dahl A., DiBernardo L., Durante-Mangoni E., Duval X. (2023). The 2023 Duke-International Society for Cardiovascular Infectious Diseases Criteria for Infective Endocarditis: Updating the Modified Duke Criteria. Clin. Infect. Dis..

[11] Hubers S.A., DeSimone D.C., Gersh B.J., Anavekar N.S. (2020). Infective Endocarditis: A Contemporary Review. Mayo Clin. Proc..

[12] Alnabelsi T.S., Sinner G., Al-Abdouh A., Marji M., Viquez K., Abusnina W., Kotter J., Smith M.D., El-Dalati S., Leung S.W. (2023). The Evolving Trends in Infective Endocarditis and Determinants of Mortality: A 10-year Experience From a Tertiary Care Epicenter. Curr. Probl. Cardiol..

[13] Seguel S.E., Rojas-Campillay C., Peralta-Jiménez G.A., Hernández-Paredes F., Vera-Calzaretta A., González L.R., Stockins L.A. (2023). Cambios en el perfil epidemiológico de la Endocarditis Infecciosa con indicación quirúrgica entre 1983 y 2020. [Changes in the epidemiologic profile in infective endocarditis with surgical indication between 1983 and 2020]. Rev. Méd. Chile.

[14] Ministerio de Desarrollo Social y Familia (CL) (2022). Encuesta de Caracterización Socioeconómica Nacional (CASEN) [National Survey of Socioeconomic Characterization (CASEN)].

[15] Urina-Jassir M., Jaimes-Reyes M.A., Martinez-Vernaza S., Quiroga-Vergara C., Urina-Triana M. (2022). Clinical, Microbiological, and Imaging Characteristics of Infective Endocarditis in Latin America: A Systematic Review. Int. J. Infect. Dis..

[16] Munera Echeverri A.G., Saldarriaga Acevedo C. (2021). Clinical, laboratory, microbiological and echocardiographic characteristics of infective endocarditis in a tertiary care hospital. Acta Med. Colomb..

[17] Habib G., Lancellotti P., Erba P.-A., Sadeghpour A., Meshaal M., Sambola A., Furnaz S., Citro R., Ternacle J., Donal E. (2019). The ESC-EORP EURO-ENDO (European Infective Endocarditis) registry. Eur. Heart J.—Qual. Care Clin. Outcomes.

